# Current status of transcutaneous auricular vagus nerve stimulation for tinnitus: a narrative review of modern research

**DOI:** 10.3389/fnins.2024.1405310

**Published:** 2024-07-04

**Authors:** Qiqi Wu, Jiawei Wang, Dexiong Han, Lala Qian, Hantong Hu, Hong Gao

**Affiliations:** ^1^Department of Acupuncture, Moxibustion and Massage, Wenzhou Central Hospital, Wenzhou, China; ^2^The Third Clinical College of Zhejiang Chinese Medical University, Hangzhou, China; ^3^Department of Acupuncture and Moxibustion, The Third Affiliated Hospital of Zhejiang Chinese Medical University, Hangzhou, China

**Keywords:** vagus nerve, tinnitus, acupoints, electrical stimulation, mechanisms

## Abstract

Tinnitus, characterized by phantom sound perception, is a highly disruptive disorder lacking definitive and effective treatments. Its intricate neural mechanisms are not fully understood. Transcutaneous auricular vagus nerve stimulation (taVNS) has demonstrated potential as a substitute or supplementary treatment by activating central vagal pathways. However, standardized therapeutic protocols and objective tests to assess efficacy are lacking. Therefore, taVNS shows promise as a therapy for tinnitus, and treatment protocols should be optimized in future clinical trials.

## 1 Introduction

The sensation of sound in the ear or head without an external sound source or electrical stimulus in the environment is known as tinnitus. It is often accompanied by adverse psychological reactions such as sleep disturbance, irritation, annoyance, lack of concentration, anxiety, and depression. Tinnitus has become one of the three major ear problems, alongside deafness and vertigo. Tinnitus is a common illness that affects 12–30% of the global population, according to epidemiological research (Gallus et al., 2015; McCormack et al., 2016); its frequency is anticipated to keep increasing, signifying an increasing worldwide burden (Langguth et al., 2013; Ali Ismail, 2023). It affects 24% of all adults, 14% of middle-aged individuals, and 10% of young adults, its frequency rises with age rather than gender (Kim et al., 2015; Jarach et al., 2022). Approximately 20% of adults with tinnitus require clinical intervention (Henry et al., 2008). The proportion of tinnitus patients is increasing, particularly among younger individuals, impacting their daily life, work, and study. In severe cases, some patients may even consider suicide.

Common risk factors for tinnitus include hearing loss, aging, and high cholesterol levels (Ali Ismail, 2023). While the exact processes causing tinnitus are still unknown, it is widely acknowledged that non-auditory brain networks, interaction between auditory and somatosensory structures, and central auditory pathways are involved in the pathophysiology of tinnitus (Langguth et al., 2019). Although various treatment options for tinnitus are available, their effectiveness is relatively low (Langguth et al., 2019). Consequently, complementary therapies (Ali Ismail et al., 2022; Ismail et al., 2022) are being researched as viable treatment options for tinnitus due to its ability to offer individualized and comprehensive care, patient empowerment regarding self-management, potential synergistic effects when combined with conventional therapies, and relative affordability.

Vagus nerve stimulation (VNS) has been used as an electrotherapy and neuromodulation technique for treating a wide range of diseases, including migraine, tinnitus, heart problems, depression, and epilepsy. In most cases, VNS is either non-invasive (using transcutaneous modalities) or invasive (including the insertion of stimulators). Transcutaneous auricular vagus nerve stimulation (taVNS) is the name used to describe non-invasive variants of this type of stimulation, when electrical stimulation is administered to the auricular branch of the vagus nerve that distributes in the concha or lower part of the rear ear (Wang et al., 2021). This perspective article, which focuses on developments in recent years in the treatment of tinnitus with taVNS, promptly highlights recent scientific work on the condition.

### 1.1 Pathogenesis and neuromodulation treatments of tinnitus

Increased understanding of tinnitus has led to the proposal of a central mechanism (Eggermont, 2015), suggesting that many forms of tinnitus are associated with hyperexcitability and impaired neural remodeling in the central auditory system. Reduced signal transduction from injured hair cells in the peripheral auditory system can result in higher synchronous firing, spontaneous activity in auditory neurons near the edge of the characteristic frequency, and diminished lateral inhibition in the central auditory system (Eggermont, 2015). Changes in the neuroplasticity of the auditory and non-auditory systems are often the cause of tinnitus. Since tinnitus results from functional alterations in neuronal activity, it is theoretically possible to suppress it by using the appropriate neuromodulation mode. Tinnitus has been treated with various neuromodulation methods, such as implantable electrical stimulation of the auditory cortex, transcranial direct current stimulation, and transcranial magnetic stimulation of the auditory cortex or cingulate gyrus (Vanneste and De Ridder, 2012). These techniques, however, cannot permanently suppress tinnitus; they can only momentarily interfere with neural activity.

### 1.2 Applications and side effects of vagus nerve stimulation treatment

Recently, VNS is an exceptional neuro-rehabilitation treatment in rats because it affects motor control and network connectivity. Pro-plasticity substances include fibroblast growth factor, norepinephrine, serotonin, acetylcholine, and brain-derived neurotrophic factor are released (Collins et al., 2021). Implanted VNS has shown promise in animal studies, suggesting that it can specifically induce remodeling of the central auditory system and potentially treat tinnitus (Engineer et al., 2011). Previous research demonstrated that VNS can reverse abnormal audio localization patterns in the primary auditory cortex, reduce frequency selectivity, and decrease synchronization of neuronal spontaneous activity, correcting impaired neural remodeling (Engineer et al., 2013). A study by De Ridder et al. revealed that VNS is beneficial for tinnitus patients and is safe and practicable when combined with tones that exclude the tinnitus-matched frequency (de Ridder et al., 2014). Even while implanted VNS has benefits, invasive intervention is unavoidably associated with risks (Ben-Menachem, 2001). Acute side effects might include numbness in the lower face, paralysis of the cocal cords, infections, and more. Hoarseness, sore throats, and voice alterations are long-term dangers (Ben-Menachem, 2002). Meanwhile, the auditory branch of the vagus nerve (ABVN) has been stimulated using a non-invasive transcutaneous device, which has been shown to produce identical functional magnetic resonance imaging (fMRI) findings with changes in brain activity compared to invasive VNS (Kraus et al., 2007). As a result, taVNS therapy has emerged as an alternative approach to replace implanted VNS for treating tinnitus.

### 1.3 taVNS activates central vagal pathways to improve tinnitus

**Figure 1:**
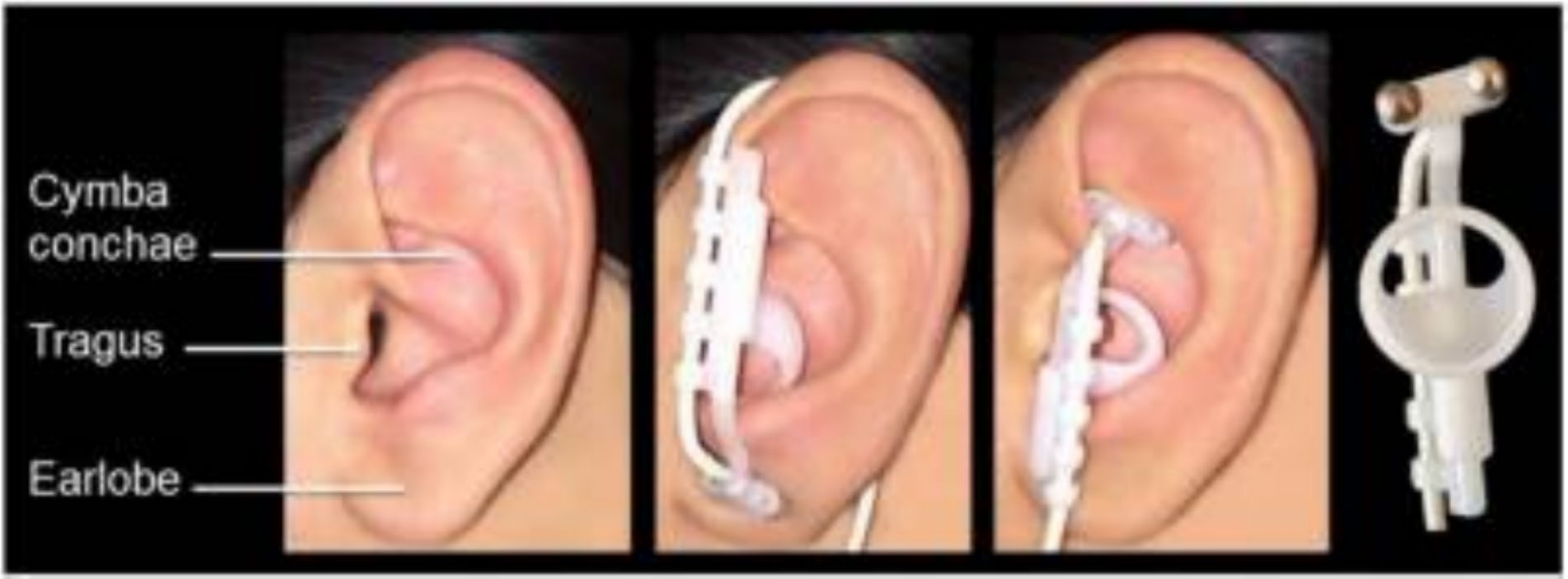
Electrode position of transcutaneous auricular vagal nerve stimulation (taVNS). Reprinted from Frangos et al. (2015).

Tenth of the twelve cranial nerves, the vagus nerve projects 80% of its fibers to the nucleus tractus solitarius (NTS) as visceral sensory afferent fibers. These fibers then project to the raphe nuclei and the locus coeruleus (LC), regulating the release of neurotransmitters like norepinephrine and acetylcholine while also inducing neuroplasticity (Manta et al., 2009; Bonaz et al., 2013). These neuromodulators influence the amygdala, hippocampus, and cortex to improve neuroplasticity. Additionally, it has been shown that acetylcholine and norepinephrine can influence the auditory cortical neurons’ selective plasticity (Manunta and Edeline, 2004; Seol et al., 2007; Edeline et al., 2011). The external auditory canal branch of the vagus nerve primarily projects to the NTS in the brainstem. TaVNS activates the ABVN for central modulation (Peter and Kleinjung, 2019; Figure 1). Two fMRI studies investigating the effects of transcutaneous electrical VNS of the tragus on human brain regions revealed changes in blood oxygen level-dependent signals primarily in the NTS, followed by the LC and the raphe nuclei regions (Kraus et al., 2007; Dietrich et al., 2008). Anatomy and imaging studies confirm that taVNS activates central vagal pathways similarly to implanted VNS (Kraus et al., 2007, 2013; Butt et al., 2020). Stimulation of the left concha auriculae also induced extensive activation along the central vagal afferent pathway, from the ipsilateral NTS to the brainstem and forebrain (Frangos et al., 2015).

### 1.4 Distinctions in our current research compared to previous reviews

Only non-invasive neuromodulation was included in the recent systematic review on the treatment of tinnitus with non-invasive nerve stimulation, which also included some studies with invasive treatment (Stegeman et al., 2021). The review included two case series, five cohort studies, and two randomized controlled trials (RCTs). In the meantime, RCTs and controlled trials that linked study quality and n-VNS’s efficacy as a tinnitus treatment with a novel method, at-VNS, were included in a systematic review and meta-analysis, which set them apart entirely from all the RCTs included in both systematic reviews (Fernández-Hernando et al., 2023). Chen et al. (2023) introduced a research review that aimed to compile ongoing research from the literature and provide an overview of the many forms of non-invasive electrical stimulation that are currently in use and their usefulness in treating tinnitus without eliminating studies based on the caliber of those studies. De Ridder et al. (2021) recently reviewed vagus nerve stimulation for tinnitus, differing completely from our perspective, which only includes taVNS. However, there is inconsistency among various literature sources regarding the site of action, stimulation technique, stimulation parameters, and other relevant factors related to taVNS. There is an urgent need for further information about the ideal taVNS parameters and methods to enable widespread clinical use. To fill up the existing research gaps, this paper summarizes recent developments in taVNS for tinnitus investigations.

## 2 Clinical research status of taVNS for tinnitus

We searched Pubmed, ScienceDirect, Web of Science, Embase (via Ovid), and Cochrane Library for relevant studies on this topic. The literature search period covers from the inception date of each database to June 14, 2024. Keywords such as “transcutaneous,” “vagus nerve stimulation,” and “tinnitus” were combined to form the basis of the search strategy. The detailed search strategy for each database is provided in Appendix S1. Notably, since studies with unpublished data are generally not peer-reviewed, to ensure higher quality in our analyzed studies, we did not search for or analyze studies with unpublished data. The main characteristics of the examined research are enumerated in Table 1.

**Table 1:** Summary of taVNS studies for tinnitus.

References	Numbers	Characteristics	Intervention and treatment	Treatment duration	Main site	Stimulation parameters	Stimulation durations	Evaluation indicators	Conclusions
Kreuzer et al. (2014)	50	Chronic tinnitus symptom duration≥6 m, TQ ≥ 31	TaVNS	24 weeks	Left tragus	0.1–10 mA, 25 Hz	4–6 h/day	THI, TQ, TBF-12, BDI, CGI-CHANGE, WHOQOL, Loudness	1. The data demonstrate the feasibility of taVNS over a period of 6 months. 2. The use of taVNS for the treatment of tinnitus cannot be recommended in its current form. 3.The data suggest taVNS to be considered safe in patients without a history of cardiac disease.
Ylikoski et al. (2020)	78	Tinnitus-related mental stress	TaVNS with standard tinnitus therapy	1 year	Left tragus	0.3–3 mA, 25 Hz	60–90 min/day, 5 days a week	The clinical features, psychophysiological characteristics, and results of the HRV tests	Tinnitus-related mental stress is an example of a stress condition in which patients may benefit from taVNS. As revealed by HRV, test-taVNS improved parasympathetic function, most efficiently in patients with a low starting HRV level. TaVNS, effectively alleviated tinnitus stress and handicap.
Tutar et al. (2020)	60	Chronic subjective tinnitus duration≥3 m	A: taVNS one ear B: taVNS both ears C: taVNS no electrical or sound stimulated	10 times	Cymba conchae	10–30 mA, 200 Hz	30 min	DASS-21, THI	TaVNS has a therapeutic effect on subjective chronic tinnitus as well as a placebo effect.
Raj-Koziak et al. (2024)	29	Chronic subjective tinnitus duration≥6 m	A: taVNS paired with sounds B: blank control	12 weeks	Left tragus	25 Hz, 250 ms	1 h	Audiological evaluation, quantitative EEG, THI, STS, VAS	The taVNS was not effective in reducing tinnitus symptoms. Changes in the theta band suggest there might be cortical effects that might, with sustained treatment, lead to improvements.
Peng et al. (2018)	24	Healthy subjects aged between 28 and 38 years	A: taVNS auricular acupoints B: taVNS anterior stimulation C: taVNS sham group	1 time	Kindey (CO10), Yidan (CO11), Liver (CO12) and Shenmen (TF4)	4–8 mA, 20 Hz	420 s	Heart rate, blood pressure, fMRI data from the cortices	TaVNS was an effective input that directly regulates the central auditory pathway. TaVNS at acupoints CO10-12, TF4 could activate the prefrontal, auditory and limbic cortices of healthy brain and this scheme could be a promising tool for tinnitus treatment.
Yakunina et al. (2018)	36	Chronic tinnitus duration≥3 m	TaVNS	6 times	Inner tragus and cymba conchae	0.1–1.8 mA, 25 Hz	5 min	FMRI to explore brain activity	TaVNS of the inner tragus and cymba conchae in patients with tinnitus successfully suppressed the auditory, limbic, and other brain areas implicated in the mechanisms involved in the generation/perception of tinnitus via auditory and vagal ascending pathways. TaVNS can potentially assist in reducing the generation and perception of tinnitus symptoms.

TQ, Tinnitus Questionnaire; TaVNS, Transcutaneous Auricular Vagus Nerve Stimulation; THI, Tinnitus Handicap Inventory; TBF-12, Tinnitus Impairment Questionnaire (TBF-12); BDI, Beck Depression Inventory; CGI, Clinical Global Impression; WHOQOL, The World Health Organization Quality of Life; HRV, Heart rate variability; DASS-21, Depression, Anxiety and Stress Scale-21; EEG, Electroencephalography; STS, The Skarzynski Tinnitus Scale; VAS, The Visual Analog Scale; FMRI, Functional Magnetic Resonance Imaging.

### 2.1 taVNS reduces tinnitus questionnaire scores, but cannot improve tinnitus complaints: an open pilot study

A study published in *Brain Stimulation* (Kreuzer et al., 2014) evaluated the viability, security, and effectiveness of taVNS for chronic tinnitus.

In detail, the study recruited 50 patients undergoing taVNS in an open single-armed pilot trial with persistent tinnitus using two distinct stimulation devices. The investigation was carried out in two parts.

The WHO Quality of Life, the TQ, the Beck Depression Inventory (BDI), the Tinnitus Handicap Inventory (THI), and several numerical rating systems were used as the basis for the clinical assessment. TQ change was identified as the primary outcome.

For the first trial phase, the primary analysis revealed substantial mean TQ reductions of 3.7 points (phase 1) and 2.8 points (phase 2). Phase 1 BDI decrease was considerable, according to secondary analyses, but no other systematic or noteworthy effects were seen. Twitching and pressure at the electrode implantation site were among the adverse effects. It was determined that the intervention had no bearing on the occurrence of a left bundle branch block and one hospitalization brought on by palpations. Tests of cognitive function revealed no significant changes.

The results show that taVNS is feasible during a six-month timeframe. However, after using taVNS for 6 months, there was no discernible improvement, which led to the conclusion that taVNS, in its current form, cannot be recommended for the treatment of tinnitus. According to data, persons without a history of heart disease can safely use taVNS.

### 2.2 taVNS attenuates tinnitus-triggered stress reaction: a retrospective study

This retrospective study (Ylikoski et al., 2020) in 171 tinnitus-related mental stress patients reports clinical parameters, psychophysiological traits, and heart rate variability (HRV) test results both before and following test-taVNS. In addition, this study presents the therapeutic effects of 113 individuals with mental stress due to tinnitus who received taVNS in addition to conventional tinnitus therapy.

Hearing and tinnitus diagnostic profiles were established. Pre- and post-stimulation HRV testing, together with test-taVNS with heart rate monitoring, were carried out to identify potential cardiac side effects. After that, daily taVNS home treatment was advised. A 1-year follow-up result was examined to evaluate the therapeutic utility of taVNS. Test results for HRV were examined in the past and linked to diagnostic information.

The majority of those experiencing mental stress linked to tinnitus also have related symptoms including anxiety and sleep difficulties. Before test-taVNS, almost 75% of the 171 individuals exhibited elevated sympathetic activity, according to baseline HRV data. In almost 80% of patients, test-taVNS changed the mean values of several HRV measures toward higher parasympathetic activity. There were no cardiac or other negative effects with test-taVNS. Surveys used to monitor the impact did not reveal any noteworthy negative outcomes.

One stress-related ailment where patients may benefit from taVNS is mental stress connected to tinnitus. Test-taVNS enhanced parasympathetic activity, as shown by HRV; this effect was particularly pronounced in individuals with low baseline HRV levels. Tinnitus stress and disability were successfully reduced by the tinnitus treatment regimen, which included taVNS. There is an urgent need for further information about the ideal taVNS parameters and methods to enable widespread clinical use.

### 2.3 taVNS has a therapeutic effect on subjective chronic tinnitus as well as a placebo effect: a randomized controlled trial

Another study published in the *American Journal of Otolaryngology* (Tutar et al., 2020) evaluated the efficacy of taVNS for treating tinnitus.

Sixty patients with persistent subjective tinnitus were included in the trial, and they were split into three groups of 20 patients each at random. TaVNS was used to stimulate one ear in the first group (A) and both ears in the second group (B). Neither sound nor electricity was applied to group (C), which served as the placebo. Ten sessions in all, with a maximum of 4 days in between, were given to each group patient.

Following the therapy, there was a substantial reduction in the THI and Depression Anxiety Stress Scale (DASS) scores (*p* < 0.05). Following therapy, a significant change was also noted between the groups (*p* < 0.05). Although group A and group B did not vary, group C’s post-treatment score was found to be considerably higher than both groups’ (*p* < 0.05).

It is noteworthy that taVNS exhibits both a placebo effect and a therapeutic impact on subjective chronic tinnitus.

### 2.4 Effectiveness of taVNS for tinnitus: an interventional prospective controlled study

A non-randomized prospective controlled trial (Raj-Koziak et al., 2024) evaluated the efficacy of taVNS combined with auditory stimulation for treating tinnitus. Over 12 weeks, 29 patients with persistent tinnitus received either taVNS with sounds (*n* = 15) or no treatment (*n* = 14). Auditory function, quantitative electroencephalography (EEG), questionnaires, and voice measures were assessed pre- and post-treatment. While subjective and objective tinnitus measures did not improve in the treatment group compared to controls, questionnaires showed statistical improvements on some parameters. Tinnitus level and frequency were similar between groups. However, the taVNS group exhibited a substantial increase in theta band activity on EEG, suggesting potential cerebral effects despite no improvement in tinnitus symptoms. The theta band alterations imply continued therapy may yield benefits.

### 2.5 taVNS can induce auditory and limbic cortices activation measured by fMRI

A different research that was published in Hearing Research (Peng et al., 2018) investigated the core mechanism of taVNS in humans using fMRI to identify a good taVNS location for the treatment of tinnitus.

Aged between 28 and 38, the research enrolled 24 healthy volunteers. In the anterior stimulation group, eight patients were stimulated at the anterior wall of the auditory canal and the left lower leg. Eight participants received stimulation at the left auricular acupoints of Shenmen (TF4), Liver (CO12), Yidan (CO11), and Kindey (CO10), while the remaining eight were placed in a sham group and were administered taVNS at the tail of the helix and left lobe.

After gathering cortical fMRI data, Alphasim analysis was carried out. They discovered that taVNS at auricular acupoints CO10–12 and TF4 may effectively and rapidly cause changes in blood oxygenation level dependent (BOLD) signals in the limbic, auditory, and prefrontal cortices of healthy people using fMRI. We found that our stimulation increased signals from the auditory ascending pathway, the prefrontal cortex, which includes the thalamus, the middle temporal gyrus, the superior temporal gyrus, and limbic system regions like the parahippocampal gyrus, amygdala, posterior cingulate cortex, caudate, and putamen when comparing the acupoints group and the sham group in the left brain. The BOLD signal between the anterior group and the acupoints group differed in the left brain’s superior temporal gyrus.

They also found differences in signal across the groups in several right brain areas. According to this study, taVNS at acupoints CO10–12 and TF4 might stimulate the limbic, auditory, and prefrontal cortices in a healthy brain, indicating that this treatment approach may be useful for tinnitus.

### 2.6 The mechanism of action of taVNS might be involved in multiple brain areas

Furthermore, the effects of taVNS on brain activity in tinnitus sufferers were investigated using fMRI (Yakunina et al., 2018). Thirty sixc patients with persistent tinnitus were given taVNS to the cymba conchae, inner tragus, and earlobe (sham stimulation).

In contrast to the sham stimulation, stimulation of both regions resulted in the activation of the brainstem’s solitary tract nucleus and locus coeruleus. Additionally, there was an activation of the cochlear nuclei, which was not seen in healthy individuals with normal hearing. The parahippocampal gyrus, which was lately postulated to be the origin of tinnitus in people with hearing impairments, was deactivated by taVNS, along with several other auditory, limbic structures and brain regions linked to the production and perception of tinnitus.

In individuals with tinnitus, taVNS via the cymba conchae or inner tragus inhibited neuronal activity in the limbic, auditory, and other non-auditory regions connected to tinnitus through vagal and auditory ascending pathways. The findings of this investigation are examined in light of several tinnitus models that are currently in use. They suggest that the mechanism of action of taVNS may include many brain regions that are in charge of producing tinnitus, causing emotional irritation associated with tinnitus, and their reciprocal reinforcing.

## 3 Discussion

There is a great deal of variation in the origin, intensity, and perception of tinnitus, which can cause anything from little irritation to severe disruptions to everyday activities (Stouffer and Tyler, 1990). Although taVNS has shown encouraging results in the therapy of tinnitus, there are insufficient high-quality studies to rule out the placebo effect.

### 3.1 Evaluating the treatment effect and mechanisms underlying taVNS

In the psychiatric domains of pain, migraine, epilepsy, depression, and tinnitus, taVNS has been extensively utilized throughout time (Wang et al., 2021). The majority of taVNS trials found that either alone or in conjunction with sound treatment, taVNS intervention was effective in suppressing tinnitus. However, a placebo effect is unavoidable when there is no sham-control design. Because nerve anatomy can be challenging, there has been a dearth of anatomical data to support the body surface distribution map of ABVN (Butt et al., 2020). Anatomical data indicates that ABVN is dispersed throughout the external auditory meatus (EAM), particularly in the EAM’s posterior wall. On the other hand, fMRI research revealed that the vagal afferent route may be activated by stimulating the anterior wall, inner tragus, and cymba concha of the EAM (Badran et al., 2018). The cavum concha, cymba concha, and inner tragus are the stimulation sites employed in the taVNS clinical research. The fMRI data can be used to mutually verify the significant clinical effects. Furthermore, Yakunina et al.’s study (Yakunina et al., 2017) indicated that the brainstem’s vagal afferent route can be most activated when the cymba concha is stimulated, suggesting that this location may be more appropriate. Nearly all taVNS investigations used suprathreshold current intensity values. The regions of interest that resulted from the current flow patterns and intensity were extremely unique to the taVNS electric montage (Kreisberg et al., 2021). Many questions remain regarding the amount and amplitude of energy given to the tissue due to the significant effect of electrode and tissue impedance, indicating that employing suprathreshold is an option (Yap et al., 2020). Only the Kreuzer et al. research (Kreuzer et al., 2014) included two cardiac events that had nothing to do with the taVNS. There is proof that taVNS is a well-tolerated and safe technique (Redgrave et al., 2018). The most frequent adverse effects include headache, nasopharyngitis, and localized skin irritation brought on by the electrode implantation. No particular therapy was needed. The outcomes of previous research on the pure use of taVNS, however, were inconsistent. It is yet unknown if it is necessary to connect the vagus stimulation with noises that are either tinnitus-matched or not (De Ridder et al., 2021). To sum up, taVNS is a workable and secure method, but there is a lot of variability in the parameter choices, which makes it difficult to synthesize and duplicate. To ensure transparency, completeness, and reproducibility, future research on taVNS should adhere to the guidelines for reporting standards (Farmer et al., 2021).

Transcallosal or cavitas conchae electrical stimulation has been shown to provide relief for tinnitus symptoms without complications or side effects. Combining this with sound stimulation can potentially result in even more effective treatment. Animal studies have established the groundwork for treating tinnitus using VNS and tones, demonstrating that VNS paired with specific frequencies can significantly increase primary auditory cortex site responses (Engineer et al., 2011). A clinical trial involving VNS and taVNS with auditory stimulation in tinnitus patients has also supported these findings (de Ridder et al., 2014).

TaVNS may serve as an adjunct treatment for tinnitus, potentially improving cognition, driving neural remodeling, and enhancing tinnitus habituation therapy (Porter et al., 2012). Ylikoski et al. found that taVNS successfully reduced disability and stress related to tinnitus by improving parasympathetic function, particularly among those whose baseline HRV level is low (Ylikoski et al., 2020).

### 3.2 The main site of stimulation for taVNS

It is important to note that while VNS stimulation on one side can modulate the other side, foreign studies predominantly stimulate the left vagus nerve due to potential cardiac complications associated with stimulating the right vagus nerve. The main site for taVNS is the tragus abroad and the cavitas conchae in China, with literature suggesting that stimulation of either site can produce similar effects to implantable VNS (Kraus et al., 2007; Dietrich et al., 2008; Fang et al., 2014). The earlobe stimulation did not show a vagus nerve-like effect (Kreuzer et al., 2014).

### 3.3 Problems and future research directions

Despite the demonstrated effectiveness of VNS for tinnitus, several issues must be addressed. First, small sample sizes limit the statistical power to determine efficacy. Uncertainty regarding treatment and stimulation durations may also affect patient compliance and the ability to reverse adverse remodeling. Inconsistent stimulation parameters make it difficult to determine the optimal combination for taVNS treatment. Studies have shown that the optimal stimulation intensity for VNS to alter cognitive function is 0.5 mA (Clark et al., 1999) and that brain regions are significantly activated when VNS is intermittently applied at 20 Hz (Lomarev et al., 2002). However, the optimal parameters of ta-VNS for the treatment of tinnitus is not known, necessitating individualized treatment plans for different patients to achieve the best therapeutic effect.

Moreover. the specificity of ear vagus nerve stimulation for tinnitus treatment should be further investigated with clinical trials including other nerve stimulations as control groups. For example, stimulating the great auricular nerve in the earlobe could help to clarify the specificity and feasibility of this method beyond the placebo effect. Objective detection methods such as salivary alpha-amylase (sAA), p300, and fMRI are lacking. In the central nervous system, sAA is collected as an indirect marker of endogenous noradrenergic activation (Ventura-Bort et al., 2018), and recent fMRI studies have shown that taVNS successfully activated the LC and NTS while reducing excitability in the auditory center and limbic system (Yakunina et al., 2017).

Differences in patient inclusion criteria may impact efficacy as well. The effect of hearing threshold on remodeling is significant since tinnitus accompanied by normal hearing or mild hearing loss does not necessarily undergo neural remodeling of the auditory cortex (Langers et al., 2012). If taVNS works by affecting neural remodeling, it may have little effect on these tinnitus patients. Therefore, future clinical trials of taVNS should categorize patients by types of tinnitus—acute, subacute, and chronic—based on the course of the disease, and further subdivide these types by the degree of hearing loss (mild, moderate, and severe) to determine the applicable range of taVNS.

## 4 Limitations of this review

Given the limited number of studies included and the considerable clinical heterogeneity (e.g., different ta-VNS treatment protocols), this study mainly uses qualitative analysis through a narrative review rather than quantitative methods such as meta-analysis. To address this, as more homogeneous trials in the future becomes available, employing meta-analytic techniques could provide more robust evidence on the efficacy of taVNS by synthesizing data from multiple studies. Additionally, future systematic reviews and meta-analyses on taVNS for tinnitus would benefit from registering protocols in registry platform such as PROSPERO to ensure transparency and adherence to systematic review guidelines.

## 5 Conclusion

TaVNS is a proven adjunctive therapy for tinnitus, primarily for the treatment of disorders associated with the main concomitant symptoms of tinnitus, such as insomnia, anxiety, and depression. TaVNS is a cost-effective, portable technique with no significant negative side effects, but it is limited by the lack of an established protocol for application (Jung et al., 2023). Although experimental studies and anatomy have been cross-referenced to suggest that the mechanism of action may be related to the vagus nerve. There is still a lack of sufficient research data to support the claim, and further animal tests and clinical studies are needed. Treatment protocols should be optimized in future clinical trials, such as expanding the sample size, establishing acoustic treatment as a control group, and applying objective examinations such as P300 and fMRI. Given that the concept of taVNS, as proposed by Ventureyra (2000), was inspired by traditional Chinese acupuncture therapy, the author believes that acupoint injection and auricular stimulation (such as auricular pressure beans, intradermal needling, and so on) should also be included in future clinical trials to compare the efficacy with electrical stimulation, screen clinically meaningful treatment protocols, provide an easy and accessible way to treat tinnitus, and organic combination in optimizing the tinnitus comprehensive treatment and diagnosis specifications.

## Author contributions

QW: Funding acquisition, Methodology, Resources, Writing – original draft. JW: Supervision, Visualization, Writing – original draft. DH: Conceptualization, Formal Analysis, Writing – review & editing. LQ: Investigation, Project administration, Writing – original draft. HH: Software, Validation, Writing – review & editing. HG: Data curation, Funding acquisition, Writing – review & editing.
