# Supplementary material for: Current status of transcutaneous auricular vagus nerve stimulation for tinnitus: a narrative review of modern research
Source: Front Neurosci. 2024 Jul 4;18:1405310. doi: 10.3389/fnins.2024.1405310 (PMC11254635; doi:10.3389/fnins.2024.1405310)
Supplement: Supplementary file 1 [file Data_Sheet_1.docx]

**Supplementary Materials: Detailed search strategies in all databases**

1. **PubMed**

#1 tinnitus [MeSH]

#2 tinnitus [Title/Abstract]

#3 #1 OR #2

#4 "taVNS"[Title/Abstract] OR "ta-VNS"[Title/Abstract] OR "VNS"[Title/Abstract]

#5 (((("vagus nerve"[MeSH Terms] OR ("vagus"[Title/Abstract] AND "nerve"[Title/Abstract]) OR "vagus nerve"[Title/Abstract] OR "vagus"[Title/Abstract]) OR vagal[Title/Abstract]) AND stimulation[Title/Abstract]) AND transcutaneous[Title/Abstract])

#6 #4 OR #5

#16 #3 AND #6

1. **ScienceDirect**

#1 tinnitus OR ear buzzing OR auditory phantom perception OR ringing in the ears OR auditory

#2 Vagus Nerve Stimulation OR VNS OR taVNS OR ta-VNS

#3 #1 AND #2

1. **Web of Science**

#1 TS= (tinnitus OR ear buzzing OR auditory phantom perception OR ringing in the ears OR auditory)

#2 TS= (Vagus Nerve Stimulation OR VNS OR taVNS OR ta-VNS)

#3 #1 AND #2

1. **Embase (via Ovid)**

#1 'tinnitus'/exp

#2 tinnitus.ab,ti OR ear buzzing.ab,tiOR auditory phantom perception.ab,ti OR ringing in the ears.ab,ti

#3 #1 OR #2

#4 'vagus nerve stimulation'/exp

#10 #3 AND #4

1. **Cochrane Library**

#1 tinnitus [Title/Abstract/Keywords] OR ear buzzing [Title/Abstract/Keywords] OR auditory phantom perception [Title/Abstract/Keywords] OR ringing in the ears [Title/Abstract/Keywords]

#2 Vagus Nerve Stimulation [Title/Abstract/Keywords] OR VNS [Title/Abstract/Keywords] OR taVNS [Title/Abstract/Keywords] OR ta-VNS [Title/Abstract/Keywords]

#3 #1 AND #2
